# Bag3-Induced Autophagy Is Associated with Degradation of JCV Oncoprotein, T-Ag

**DOI:** 10.1371/journal.pone.0045000

**Published:** 2012-09-12

**Authors:** Ilker Kudret Sariyer, Nana Merabova, Prem Kumer Patel, Tijana Knezevic, Alessandra Rosati, Maria C. Turco, Kamel Khalili

**Affiliations:** 1 Department of Neuroscience and Center for Neurovirology Temple University School of Medicine, Philadelphia, Pennsylvania, United States of America; 2 Department of Pharmaceutical and Biomedical Sciences (FARMABIOMED), University of Salerno, Fisciano, Italy; University of Nebraska - Lincoln, United States of America

## Abstract

JC virus, JCV, is a human neurotropic polyomavirus whose replication in glial cells causes the fatal demyelinating disease progressive multifocal leukoencephalopathy (PML). In addition, JCV possesses oncogenic activity and expression of its transforming protein, large T-antigen (T-Ag), in several experimental animals induces tumors of neural origin. Further, the presence of JCV DNA and T-Ag have been repeatedly observed in several human malignant tissues including primitive neuroectodermal tumors and glioblastomas. Earlier studies have demonstrated that Bag3, a member of the Bcl-2-associated athanogene (Bag) family of proteins, which is implicated in autophagy and apoptosis, is downregulated upon JCV infection of glial cells and that JCV T-Ag is responsible for suppressing the activity of the BAG3 promoter. Here, we investigated the possible impact of Bag3 on T-Ag expression in JCV-infected human primary glial cells as well as in cells derived from T-Ag-induced medulloblastoma in transgenic animals. Results from these studies revealed that overexpression of Bag3 drastically decreases the level of T-Ag expression by inducing the autophagic degradation of the viral protein. Interestingly, this event leads to the inhibition of JCV infection of glial cells, suggesting that the reduced levels of T-antigen seen upon the overexpression of Bag3 has a biological impact on the viral lytic cycle. Results from protein-protein interaction studies showed that T-Ag and Bag3 physically interact with each other through the zinc-finger of T-Ag and the proline rich domains of Bag3, and this interaction is important for the autophagic degradation of T-Ag. Our observations open a new avenue of research for better understanding of virus-host interaction by investigating the interplay between T-Ag and Bag3, and their impact on the development of JCV-associated diseases.

## Introduction

JCV is a human polyomavirus that infects greater than 70% human population during childhood, and establishes a latent infection for the rest of life in healthy individuals [1], [2]. Reactivation of the neurotropic strain of JCV and its replication in glial cells leads to the development of the fatal demyelinating disease of the CNS, progressive multifocal leukoencephalopathy (PML), which is seen in immunocompromised patients, notably AIDS patients [3], [4], [5]. Recently, increasing cases of PML have also been reported in patients with autoimmune diseases who have been treated with immunomodulatory regimens including Natalizumab, Rituximab, and Efalizumab [6], [7], [8], [9]. Similar to other polyomaviruses, the product of the viral early genome, T-antigen, plays a critical role in orchestrating the various stages of the viral lytic cycle including DNA replication, late gene activation, and regulation of its own promoter activity. JCV T-antigen has transforming activity and regulates several cellular events associated with control of cell proliferation, differentiation, and apoptosis [10]. For example, T-Ag binds to and inhibits the activities of several tumor suppressor proteins such as p53 and members of the retinoblastoma (pRB) family [11]. Furthermore, T-Ag induces cell growth by interacting with cellular transcription pre-initiation complexes, binding to cellular DNA, DNA polymerase α, and ATPase-helicase [12], [13]. In a previous study we demonstrated that JCV T-Ag inhibits expression of Bag3, a member of the Bag, Bcl-2-associated athanogene) family of molecular co-chaperone proteins [14], during the course of productive viral infection of glial cells by suppressing transcription of the Bag3 promoter [15]. Bag3 was initially discovered based on its binding ability to Bcl-2 [16] and has been implicated as a modulator of cellular responses to stress by interacting with the ATPase domain of Hsc70/Hsp70, and suppressing the chaperone activity of the complex [17]. Expression of Bag 3 is induced by stress-inducing agents such as high temperatures and heavy metals and by viral infection including HIV-1 [14], [18]. In addition, recent studies have demonstrated that down regulation of Bag3 sensitizes primary microglial cells to caspase-3 activation following HIV-1 infection, suggesting a unique role for Bag3 in the interaction of HIV-1 with host cells [19]. Moreover, Bag3 is shown to be overexpressed in different types of tumors including glioblastoma and has been implicated as a tumor pro-survival factor [14], [20], [21].

Here we report a novel role for Bag3 in impacting the stability of the JCV T-Ag, thus controlling the JCV lytic cycle and its interaction with host cells. A series of molecular studies suggest that Bag3 interacts with T-Ag and its overexpression downregulates T-Ag levels by inducing autophagic degradation of viral protein. Our observations ascribe a new role for Bag3 in controlling the life cycle of JCV and the progression of PML by downregulating T-Ag expression, and determining the oncogenic potential of JCV by decreasing the levels of its transforming protein.

## Results

### Bag3 Inhibits Expression of JCV T-antigen

**Figure 1:**
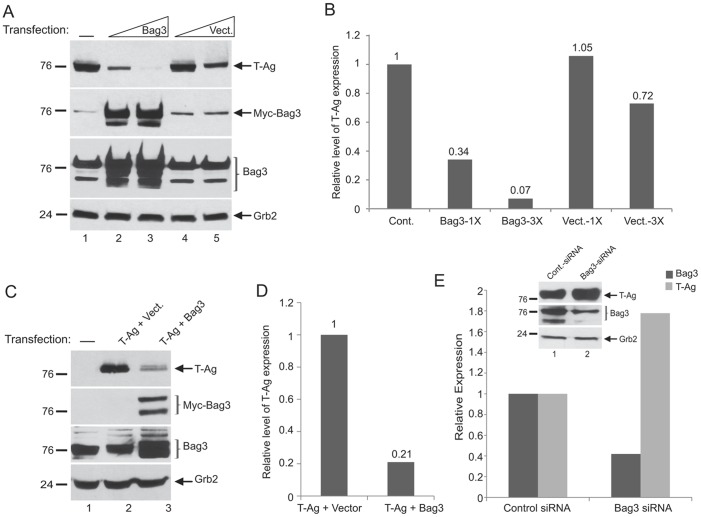
Effect of Bag3 on T-Ag expression in JCV transformed cells. A. BSB8 cells were transfected with empty vector or with an expression plasmid encoding Bag3 in a dose dependent manner. Western blot analyses were performed using whole cell protein extracts prepared at 48 hour post-transfections using specific antibodies recognizing T-Ag, Myc-tag, and Bag3 proteins. Western blot analysis of the same extracts using anti-Grb2 antibody was used as loading control. B. Relative intensity of the bands corresponding to T-Ag from panel A determined by densitometry is shown as a bar graph. C. Western blot analyses of protein extracts from U-87MG cells after transfection with plasmids encoding T-Ag and Bag3 using specific antibodies for T-Ag, Myc-tag, and Bag3 proteins. Western blot analyzsis of same extracts with anti-Grb2 antibody was used as loading control. D. Relative intensity of the bands from experiments illustrated in Panel C are shown as a bar graph. E. BSB8 cells were transfected with a Bag3 siRNA or a control non-targeted siRNA and expression of T-Ag and Bag3 was examined by Western blot analysis. Relative intensity of the bands corresponding to T-Ag and Bag3 were densitometrically determined and normalized to those from Grb2, and illustrated by a bar graph.

As a first step to investigate the impact of Bag3 on expression of JCV T-Ag, we used BSB8 as a cell culture model. These cells are derived from primitive neuroectodermal tumors that were developed upon expression of T-antigen of JCV in transgenic mice and constitutively produce T-antigen [22]. As seen in Fig. 1A and B, overproduction of Bag3 in BSB8 cells by a plasmid expressing Bag3 caused a drastic decrease in the level of T-Ag. Under identical conditions, control cells that received empty plasmid showed no significant changes in the level of T-Ag. We also utilized the human astrocytoma cell line, U-87MG, to evaluate the effect of overexpressed Bag3 on T-Ag levels that are produced upon transfection of U-87MG with a plasmid expressing JCV T-Ag under the control of the CMV promoter. Results from this experiment also showed a substantial decrease in the level of T-Ag in cells co-transfected with plasmids encoding Bag3 and T-Ag (Fig. 1C and D). These observations suggest that overexpression of Bag3 severely diminishes the level of T-Ag that is produced either endogenously by integrated transgene in chromosomes or ectopically upon transient transfection of cells with expression vector. To further establish the negative effect of Bag3 on production of T-Ag and demonstrate the direct impact of Bag3 on T-Ag levels BSB8 cells were transfected with either Bag3 specific siRNA or the control non-target siRNAs, and the level of T-Ag was determined. Results from this experiment showed that a deacrease in the level of Bag3 leads to increased levels of T-Ag in the cells treated with Bag3 siRNA, but not the control siRNA (Fig. 1E). These observations, once again, verified the negative impact of Bag3 upon T-antigen expression.

**Figure 2:**
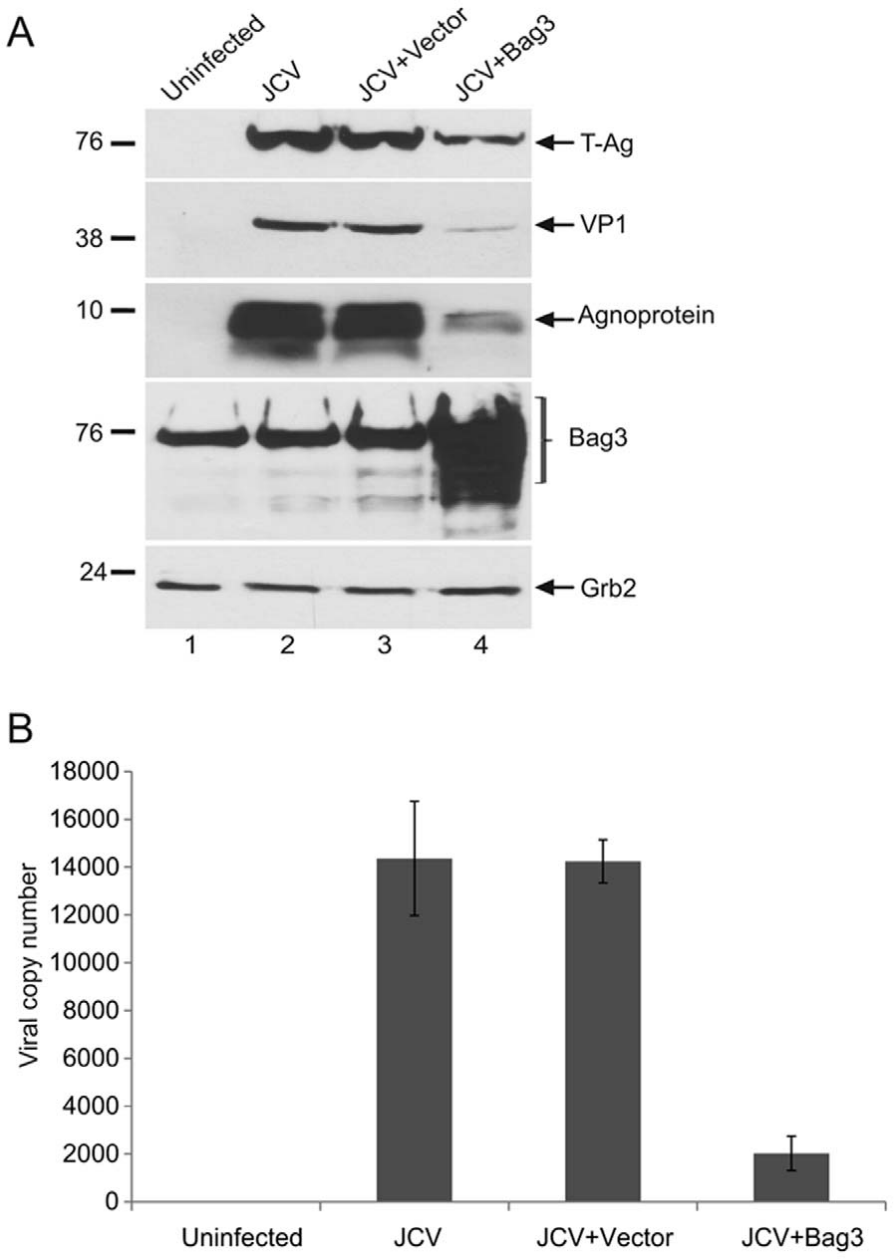
Bag3 inhibits replication of JCV in primary culture of human fetal glial cells. A. Western blot analyses of whole cell protein extracts prepared from JCV infected cells using specific antibodies against T-Ag, VP1, Agnoprotein, Bag3, and the housekeeping protein, Grb2. In lane 1, extracts from uninfected cells were loaded as a control. B. Q-PCR analyses of the viral particles in the JCV infected-cells culture medium as described in the Materials and Methods.

In the next series of experiments, we investigated the impact of Bag3 on expression of T-Ag during the course of JCV infection of glial cells. We utilized primary culture of human fetal glial cells, a culture system that supports expression of the JCV genome and its lytic infection cycle. Cells were transfected with full-length DNA of the Mad-1 strain of JCV either alone or together with Bag3 expression plasmid, and the level of T-Ag was determined by gel analysis. As seen in Fig. 2A, a noticeable decrease in the level of T-antigen was observed in cells that received JCV genomic DNA along with Bag3 expression plasmid in comparison to those seen in the control cells. As T-Ag is critical for stimulating viral late genes encoding capsid proteins including VP1 and the non-structural agnoprotein, we also examined the level of these proteins in the infected cells. As shown in Fig. 2A, a substantial decrease in the level of VP1 and agnoprotein was observed upon the overexpression of Bag3. Examination of viral load by quantitative PCR in culture media verified a greater than 7-fold decrease in the viral DNA copy number when Bag3 was overexpressed in the cells. These observations suggest that a decrease in the level of T-Ag by Bag3 has a functional consequence and negative impact on propagation of JCV in the infected cell culture system.

### Post-transcriptional Suppression of T-Ag by Bag3 is Independent from Proteasomal Pathway

**Figure 3:**
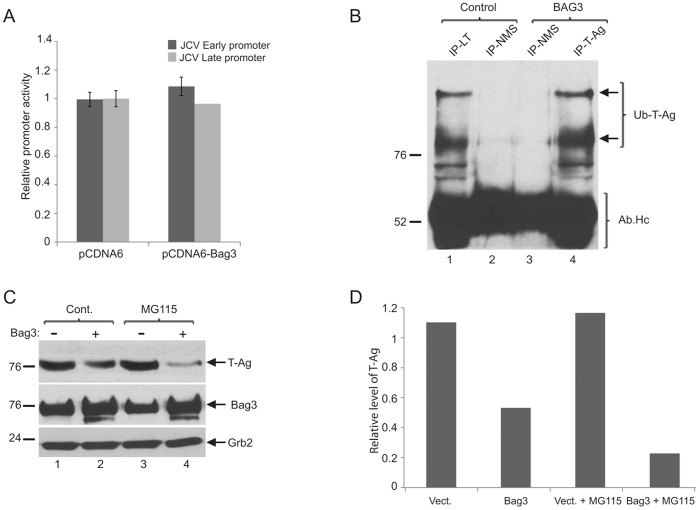
Effect of proteasomal inhibitors on Bag3-mediated downregulation of Large T antigen expression. A. Effect of Bag3 on transcription of JCV promoters. Primary culture of human fetal glial cells was transfected with CAT reporter genes under the control of JCV early or late gene promoters along with a plasmid expressing Bag3 (pCDNA6-Bag3), or a control empty vector (pCDNA6). Relative promoter activity was determined as described in the Materials and Methods. B. Protein extracts were prepared from BSB8 cells transfected with plasmid vector (control) or expression plasmid encoding Bag3. Large T-Ag was immunoprecipitated with a monoclonal antibody, separated on SDS-PAGE gel, and processed for Western blotting using anti-Ubiquitin antibody. Normal mouse serum (NMS) was used as negative control for immunoprecipitations. C. BSB8 cells were transfected with an expression plasmid encoding Bag3, and treated with MG-115 or DMSO (control). Whole cell extracts were prepared at 48 hour post-transfection, and Western blot analysis was performed for the detection of Large T, and Bag3 proteins. Western blot analysis of the same extracts with anti-Grb2 antibody was used as loading control. D. Relative band intensity of T-Ag bands from Panel B is shown as a bar graph.

To gain more insight into the mechanism by which Bag3 causes a decrease in the level of T-Ag, we performed transcription assay utilizing a CAT reporter plasmid under the control of the JCV early promoter. Results of this experiment ruled out the effect of Bag3 on transcription of the viral promoter, as no significant differences in the level of CAT activity was observed upon Bag3 overexpression (Fig. 3A). This observation led us to believe that Bag3 mediates its negative effect on T-Ag expression, at least in part, at the post-trancriptional level. One of the major pathways that is responsible for protein turnover is cytosolic destabilization of proteins through proteasomal degradation. In order to test the possible involvement of Bag3 in proteasomal degradation of T-Ag, we sought to determine the level of T-Ag ubiquitination by co-immunoprecipitation of this protein in the control cells and in cells overexpressing Bag3. Selective linkage of the chain of ubiquitins to the proteins usually leads to the degradation of the protein of the proteasomes. As seen in Fig. 3B, overexpression of Bag3 in BSB8 cells showed no significant change in ubiquitination of T-Ag in comparison to those seen in control cells (compare lane 4 with lane 1). Treatment of BSB8 cells with MG115, a potent reversible proteasome inhibitor failed to rescue degradation of T-Ag even in cells overexpressing Bag3 (Fig. 3C and D), suggesting that, Bag3 suppression of T-Ag levels is independent from the proteasomal degradation pathway.

### Bag3 Induces Autophagy and Degradation of T-Ag

**Figure 4:**
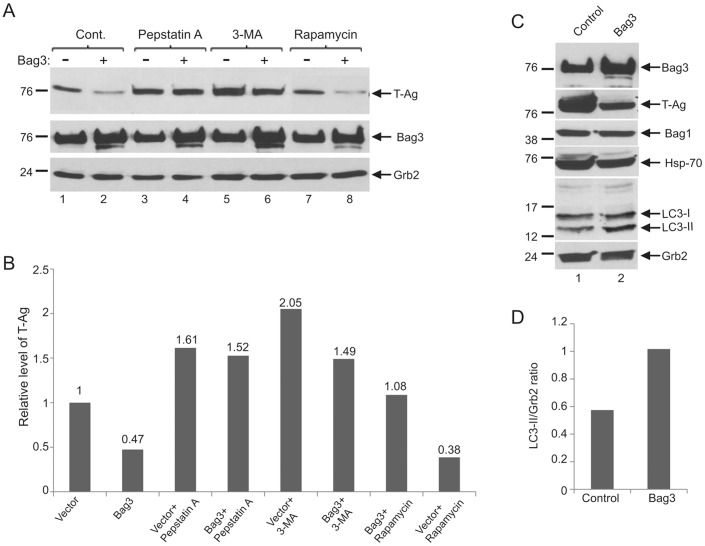
Autophagic degradation of large T antigen is induced by Bag3. A. BSB8 cells were transfected with an expression plasmid encoding Bag3, and treated either with pepstatin A, 3-MA, or rapamycin. Whole cell protein extracts were prepared at 48 hour post-transfection, and Western blot was performed for the detection of T-Ag, and Bag3 proteins. Western blot analysis of the same extracts with anti-Grb2 antibody was used as loading control. B. Relative intensity of the band corresponding to T-Ag from Panel A is shown as a bar graph. C. BSB8 cells were either transfected with control empty plasmid (lane 1) or expression plasmid encoding Bag3 protein (lane 2). Protein extracts were prepared at 48 hour post-transfection, and Western blot analysis was performed for the detection of Large T, Bag3, Bag1, Hsp70, LC3, and Grb2 proteins. D. Relative intensity of the bands corresponding to LC3-II seen in Panel C were determined by densitometry and after normalization to that from Grb2, illustrated as a bar graph.

In addition to proteasomes, the second major pathway that participates in the control of protein stability and its degradation is the lysosomal pathway which involves proteases in acidic organelles. Bag3 has been suggested to play a role in regulation of autophagy in different pathologic conditions leading to ER stress [23], [24], [25], [26]. To test a possible role for Bag3 in lysosomal degradation of T-Ag, BSB8 cells transfected with either vector alone or plasmid expressing Bag3 were treated with pepstatin A, a potent acid protease inhibitor, 3-MA, which inhibits autophagy by blocking autophagosome formation via the inhibition of type III Phosphatidylinositol 3-kinases (PI-3K), or rapamycin, which induces autophagy by suppressing mTOR. As shown in Fig. 4 (Panels A and B), treatment of the cells with pepstatin A and 3-MA blocked the ability of ectopically expressed Bag3 to decline T-Ag production. It was also noted that these treatments modestly improved T-Ag expression in the untransfected cells, suggessting that expression of T-Ag is continuously under a negative control by the baseline level of endogenous Bag3 in these cells. This observation corroborates the results from siRNA studies shown in Fig. 1E in which knock down of the endogenous Bag3 by siRNA elevated the level of T-Ag in the cells. Treatment of cells with rapamycin had no significant effect on the level of T-Ag in control and in cells with overexpression of Bag3 (compare lanes 1 and 2 with lanes 7 and 8). Our results also show that overexpression of Bag3 leads to increased levels of LC3-II, an activated form of microtubule-associated protein light chain 3 (LC3), which is associated with autophagosomal membrane (Fig. 4C and D). Altogether, these results suggest that overexpression of Bag3 induces degradation of large T-Ag through the lysosomal pathway in JCV transformed cells. Moreover, our results show that expression of Bag3 has no significant effect on the level of other members of the Bag family including Bag1 and their partners including Hsp70 (Fig. 4C).

### Bag3 Interaction with T-Ag Requires Zinc Finger Domain

**Figure 5:**
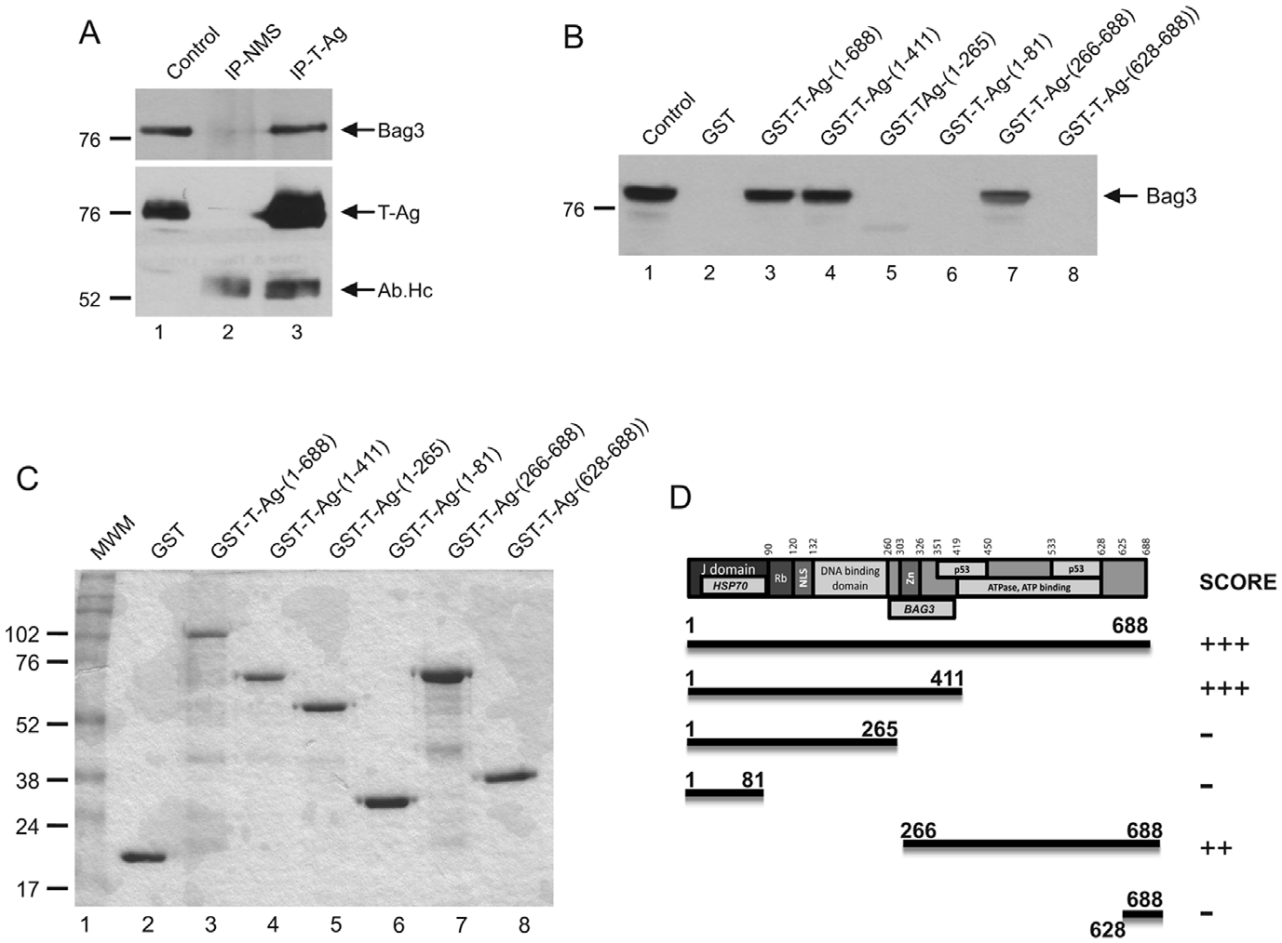
Mapping of Bag3 binding site within T-Ag. A. Bag3 was co-immunoprecipitated with a large T-Ag specific antibody from BSB8 cells as described in Materials and Methods. The Ab.Hc arrow points the heavy chain of the large T-Ag antibody. In lane 1, positive control was whole cell extracts from BSB8 cells used for IPs. B. For GST-pull down assay, full-length T-Ag and its mutant forms were produced in bacteria cells in fusion with GST and after purification, they were incubated with protein extracts prepared from U-87MG cells. Immunocomplexes were separated on a SDS-PAGE and the association of Bag3 with each protein extract was determined by Western blot analysis using a Bag3 specific antibody. C. Coomassie blue staining of bacterially produced protein used in GST-pull down assay. D. Schematic presentation of T-Ag with its various domains and scoring of Bag3 interaction with various T-Ag mutant proteins.

In light of the earlier observations on the physical association of T-Ag with Hsc70/Hsp70 and Bag3 interaction with Hsc70/Hsp70 [27], we initiated a series of studies to evaluate the ability of Bag3 for interaction with T-Ag and the importance of this interaction in the stability of T-Ag. As shown in Fig. 5A, results from co-immunoprecipitation experiments showed that the immunocomplex pulled down with anti-T-Ag antibody contains Bag3, pointing to the association of T-Ag with Bag3. In order to determine the region(s) within T-Ag that interact with Bag3, full-length T-Ag and it various deletion mutants, as illustrated in Fig. 5D, were expressed in bacteria as fusion protein to GST (Fig. 5C) and used in GST-pulldown assays according to the procedure described in Materials and Methods. Results from these experiments indicated that Bag3 recognizes a specific region within T-Ag, which spans amino acids 266–411 (Fig. 5B). This region of T-Ag has a zinc finger structure and partially overlaps with the p53 binding domain of T-Ag (Fig. 5D). Of note, this region is distinct from the prevoiusly identified J-domain of T-Ag that associates with Hsc70/Hsp70.

### Bag3 Interacts with Large T antigen through its Proline-rich Domain

**Figure 6:**
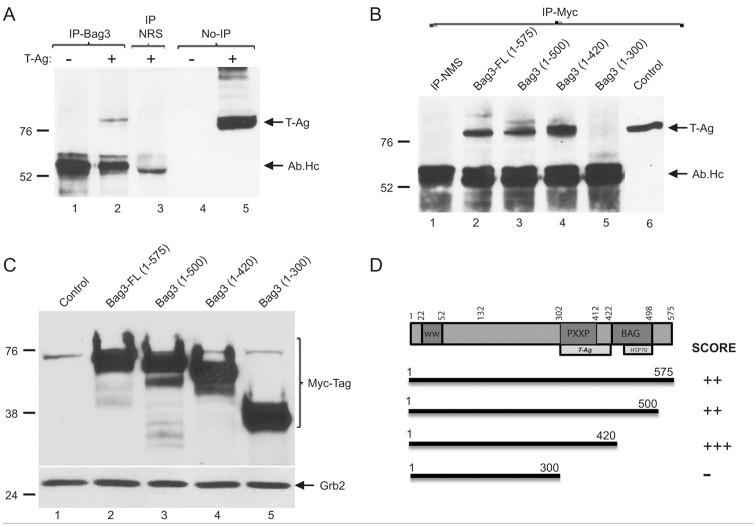
Identification of T-Ag binding site within the Bag3 protein. A. Co-immunoprecipitation of Bag3 with T-Ag. Protein extracts from U-87MG cells transfected with T-Ag expression plasmid were treated with anti-Bag3 antibody or normal sera (NRS), and the immunocomplexes were analyzed by Western blot using anti-T-Ag antibody. B. Mapping analyses of Bag3 domains that interact with T-Ag. U-87MG cells were transfected with expression plasmids encoding T-Ag along with plasmids expressing full-length Bag3 or its mutant forms. Protein extracts were immunoprecipitated by an anti-myc tag antibody that pulls down the Bag3 fusion proteins. Immune complexes were separated on a SDS-PAGE gel, and Western blot assay was performed to using anti-T-Ag antibody. Antibody heavy chain (Ab.Hc) was also detected by the large T-Ag antibody and is shown as Ab.Hc. C. Western blot analysis for detection of Bag3 full-length protein and its mutant forms using myc-tag antibody as described in Panel B. D. Schematic presentation of the various domains of Bag3 and its binding site to T-Ag.

In reciprocal experiments, to confirm the interaction between T-Ag and Bag3, and to determine the T-Ag binding site within Bag3, U-87MG cells were transfected with JCV T-Ag expression plasmid and total protein extracts from the cells were used for binding assay. Results from co-immunoprecipitation assay showed that the immunocomplex pulled down by anti-Bag3 antibody contains T-Ag antigen (Fig. 6A). Bag3, which was initially identified based on its binding ability to the ATPase domain of Hsp70, contains various regions including the WW domain at the N-terminus, a proline-rich repeat (PXXP) that mediates distinct physiological functions in the cell through interactions with other binding partners of the protein, and the Bag3 domain and the C-terminus that binds to Hsp70 [17], [26], [28]. In order to determine the T-Ag binding site within the Bag3 protein, BSB8 cells were transiently transfected with plasmids encoding full-length or mutant forms of Bag3 in fusion with myc/his tag at their C-terminus region (Fig. 6C). Bag3 and its mutant forms were immunoprecipitated with an anti-myc antibody, and immune complexes were analyzed by Western blot using an anti-T-Ag antibody. As shown in Fig. 6B and D, the full-length Bag3 and its mutants spanning the amino acid regions 1 to 500 and 1 to 420 associated with T-Ag, whereas the mutant Bag3 which lacks the PXXP domain lost its ability to interact with T-Ag. This experiment identified the PXXP region of Bag3 as a domain for its interaction with T-Ag.

### Importance of Proline-rich Domain of Bag3 in Expression of T-antigen

**Figure 7:**
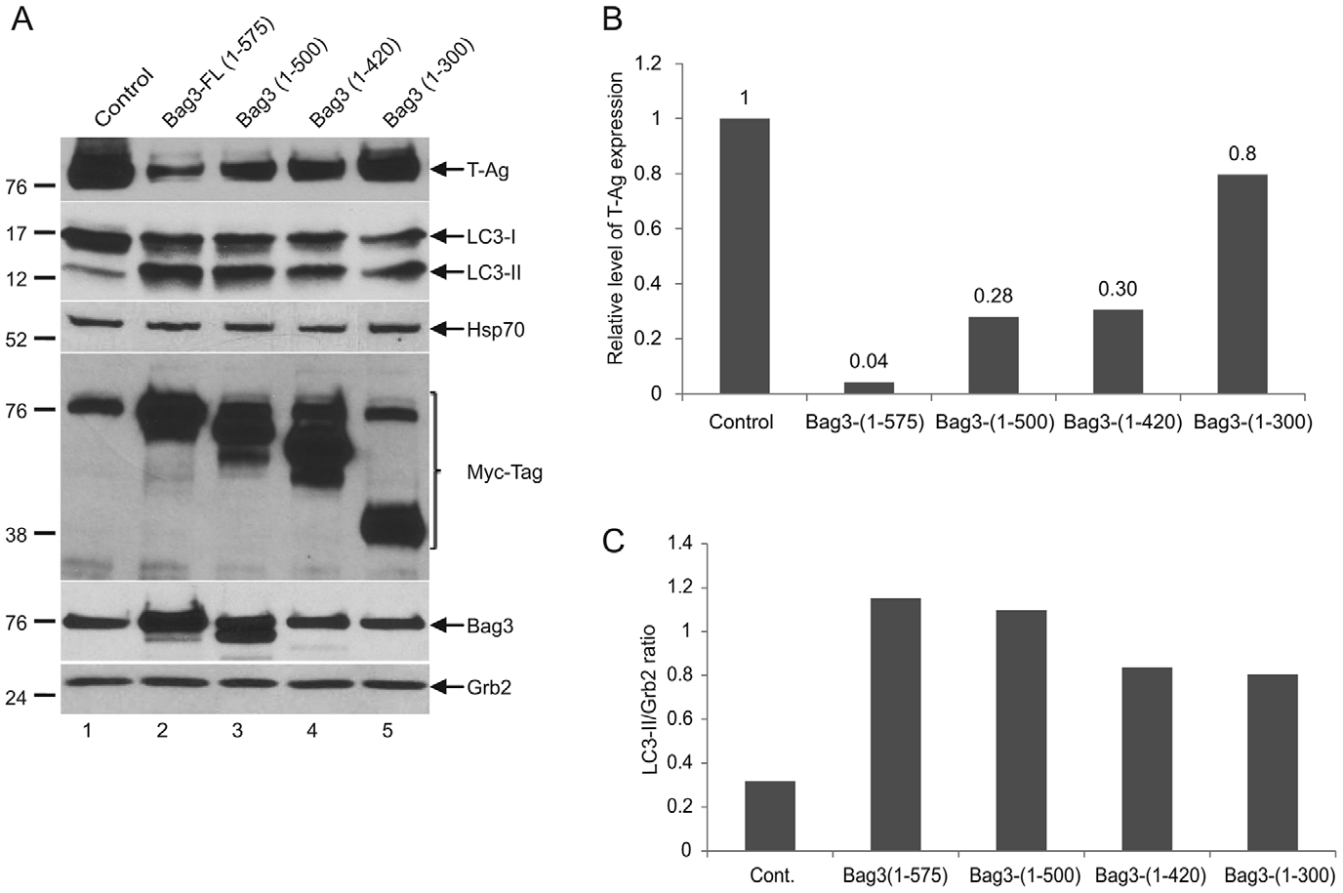
Role of the proline-rich domain of Bag3 in the inhibition of T-Ag. A. Effect of Bag3 mutant proteins on the stabiliy T-Ag. BSB8 cells were transfected with either pCDNA6 Myc-His-Bag3 FL (full-length) expression plasmid (lane 2) or its mutants (lanes 3–6), and after 48 hours, proteins were prepared and analyzed by Western blot using anti-T-Ag, anti-LC3, anti-Hsp70, anti-Bag1, anti-Myc-tag anti-Bag3, and anti-Grb2 antibodies. The control was examination of protein extracts from cells transfected with pCDNA6 Myc-His vector alone (lane 1). B. The relative intensity of the band corresponding to T-Ag from Panel A is shown as a bar graph. C. Relative intensity of LC3-I and LC3-II bands from panel A are determined and LC3-II/Grb2 ratio is shown as a bar graph.

Our studies regarding the functional interaction between large T-Ag and Bag3 suggest that Bag3 negatively regulates the level of T-Ag by inducing the autophagic degradation of this protein. Moreover Bag3 forms a complex with T-Ag through its PXXP domain. To investigate the possible role of PXXP and the other domains of Bag3 in the control of T-Ag expression, BSB8 cells were transfected with various plasmids encoding full-length and several mutant forms of Bag3 protein in fusion with myc/his tags at the C-terminus. Results from protein analysis revealed that the PXXP domain which was required for Bag3-T-Ag interaction, is critical for declining the level of T-Ag expression in the cells (Fig. 7A and B). In order to test the importance of the PXXP domain in autophagic degradation of large T-Ag, again we examined the level of LC3, a known autophagy marker, in the cells expressing the various mutants of Bag3. LC3-I was effectively converted (conjugated) in cells expressing full-length and mutant variants (Fig. 7A and C), suggesting that autophagy was induced by full-length Bag3 and its various mutants including the mutant Bag3 that lacked the PXXP domain. This observation suggests that the PXXP domain, which is required for interaction of Bag3 with T-Ag, is important for autophagic degradation of T-Ag. As seen in Fig. 7A, the level of Hsp70 remained virtually unchanged upon expression of full-length Bag3 or its various mutants. Similarly, expression of full-length Bag3 or its mutant forms showed no impact on the level of expression of Hsc70/Hsp70 in BSB8 cells.

## Discussion

To accomplish its regulatory function upon various stages of the JCV lytic cycle, the early protein of JCV, T-Ag, directly and indirectly communicates with various cellular proteins. The data presented in this study suggest that the Bcl-2-associated athanogene, Bag3 has the ability to interact with T-Ag and downregulate the level of T-Ag expression in the cells. While our initial speculation was that suppression of viral gene transcription by Bag3 is responsible for the low level of T-Ag production in cells overexpressing Bag3, results from transcription experiments showed no significant impact of Bag3 on viral early promoter activity.

Thus, it became clear that Bag3 influences expression of T-Ag at the post-transcriptional level, most likely by destabilizing the protein. Bag3 has been implicated in intracellular protein quality control by facilitating degradation of misfolded/non-functional protein, which may have a toxic effect on cells. Results of our infection studies clearly reveal that overexpression of Bag3 inhibits expression of T-Ag, which in turn, negatively impacts the transcriptional activation of the viral late gene and virion formation. Thus, the impact of Bag3 upon JCV T-Ag expression and its lytic infection may be considered as a host defense mechanism to halt the infection process. Bag3 had no effect on the state of T-Ag ubiquitination suggesting that the turnover of T-Ag may not, at least fully, be regulated by a proteasomal degradation pathway. Furthermore, treatment of cells with MG115, a proteasome inhibitor, failed to rescue T-Ag from Bag3-induced degradation, hence an alternative pathway distince from the proteasomal pathway is involved in destabilization of T-Ag. Accordingly, by using several inhibitors that affect autophagy including pepstatin A, we found that Bag3, by inducing autophagy, causes T-Ag degradation via a lysosomal pathway. With the notion that these treatments may exert a toxic effect on the cells, we performed cell viability experiments including MTT assay and found no noticeable deleterious effect of the inhibitors on cells under our experimental conditions. Indeed, more comprehensive studies that include genetic ablation of the various autophagy-associated genes, singly or in combination, can further shed light on our current observations (in progress). To further investigate Bag3 and T-Ag interplay, we investigated the possible physical interaction of these two proteins and results of our study identified a specific segment of T-Ag encompassing the zinc finger motif and part of the p53 binding site, amino acids 266–411, an important region for Bag3:T-Ag interaction. Whether or not Bag3 directly binds to this region of T-Ag or specific configuration of this domain of T-Ag, and/or its oligomerization supports interaction of T-Ag with Bag3, requires large scale mutational studies including amino acid substitution of the zinc-binding sequence. Also, it is of interest to determine whether interaction of Bag3 with T-Ag has any impact on T-Ag association with p53, a known event that influences the transforming activity of T-Ag. Our reciprocal mutational studies led to the identification of the proline-rich domain, PXXP, of Bag3, which is located in the middle of the protein as a site for T-Ag interaction. Our several efforts to express only the PXXP domain in transfected cells and evaluate its interaction, independent from the other regions of Bag3, with T-Ag was not successful, leading us to believe that this region of Bag3 is highly unstable in the cells. We also found that mutant Bag3 with a deletion in the PXXP domain is capable of inducing autophagy, yet not able to interact with T-Ag and affect its stability. This was a surprising observation that led us to speculate that the interaction of T-Ag with Bag3, through the PXXP domain, coupled with induction of autophagy by Bag3 is critical for destabilization of T-Ag in the cells. Indeed, other cellular proteins including HSPB8, which has been show to interact with Bag3 and implicated in autophagy [29], may also participate in the interplay between Bag3 and T-Ag. In addition, Hsc70/Hsp70, which exhibits binding affinity to both T-Ag, through its J-domain at the N-terminus of the protein, and to Bag3, via the Bag domain located in the C-terminus of the protein [27], [30], [31] by forming a ternary complex with Bag3 and T-Ag and/or control Bag3 association with T-Ag and the stability of the viral protein in cells. On the other hand, Bag3 may play a role as co-chaperone between T-Ag and Hsc70/Hsp70 or targets T-Ag as a client that may induce autophagic process in cells. Further investigation that includes participation of various T-Ag and Bag3 partners will provide new insight into the pathways involved in the control of viral regulatory proteins and their effect upon viral and host cells.

## Materials and Methods

### Ethics Statement

Cultures of primary human fetal astrocytes were prepared from human fetal brain tissue obtained under approval of the Temple University Institutional Review Board (IRB). The study was classified as exempt (category 4) by the IRB, and therefore, per IRB guidelines, a waiver of consent was approved and no informed consent was required or obtained.

### Cell Lines

The human malignant glioma cell line, U-87 MG, was obtained from the American Type Culture Collection (ATCC), and was grown in Dulbecco’s Modified Eagle’s Medium (DMEM) containing 10% heat-inactivated fetal bovine serum (FBS) and penicillin/streptomycin (100 µg/ml). Primary human fetal glial cells were prepared as previously described [32]. Briefly, human fetal brain tissue was obtained from Advanced Biosciences Resources, Inc. (Alameda, CA). The tissue was washed with HBSS medium and placed in a 100 mm dish. Blood vessels and meninges were dissected, and tissue was cut into small pieces using a forceps and scalpel. Chopped-tissue was mechanically disrupted by pipetting up and down in HBSS with a 10 ml pipette until cell culture fluid smooth and pinkish in color. The tissue was centrifuged and digested with DNAse I and trypsin in 10 ml HBSS medium for 30 minutes at 37°C. Cells were washed with HBSS and passed through a 70-micron filter. Mixed cultures of glial cells were plated in Poly-D-Lysinized T162 cm 2 flasks with DMEM/F12 medium (1/1) containing 10% FBS, 1% L-glutamine, 1% Fungizone, insulin, and gentamycin. After plating 4–5 days, the cells were washed with PBS and trypsinized. They then were plated in T162 cm 2 flasks and incubated for 45 minutes. During the 45 minute period, microglial cells attached to the flask, and most of the astrocytes, neurons, and oligodendrocytes remained in the medium. After 45 minute incubation, the medium was removed and placed in new flasks. The cells were grown in culture until they were confluent. Once confluent, the cells were placed on an orbital shaker to remove the neurons and oligodendrocytes, which detached from the surface of the flasks and came off into the medium. After proper shaking, the medium was replaced with astrocyte growth medium, DMEM/F12 (1/1) with 15% FBS, 1% L-glutamin, insulin, and gentamycin. BsB8 cells were derived from primitive neuroectodermal tumors that developed in transgenic mice expressing the early genome of the JC virus [22].

### Plasmids and Reagents

Fugene 6 was purchased from Roche (Basel, Switzerland). Bradford Reagent was from Bio-Rad. Polyclonal anti-BAG3 antibody was from Proteintech Group (Chicago, IL). Monoclonal anti-Myc Tag (9B11)\from Cell Signaling (Danvers, MA). Anti-Grb2 was purchased from Cell Signaling. Anti-Hsp70 (SPA810) was purchased from Stressgen-Assay Design (Ann Arbor, MI). Anti-ubiquitin (P4D1) was from Santa Cruz (Santa Cruz, CA). Protein A/G mix was from Invitrogen. Goat anti-(mouse IgG)-peroxidase conjugate and goat anti-(rabbit IgG)-peroxidase conjugate were from Pierce. pcDNA6 Myc-His-Bag3WT-(1–575), pcDNA6 Myc-His-BAG3-(1–500), pcDNA6 Myc-His-BAG3-(1–420), and pcDNA6 Myc-His-BAG3-(1–300) were reported previously [27], [33], [34]. The glutathione S-transferase (GST) fusion protein of JCV T antigen pGEX2T-LT-(1–688) and its C-terminal [pGEX2T-LT- (1–411), pGEX2T-LT- (1–265), and pGEX2T-LT- (1–82)] and N-terminal [pGEX2T-LT- (266–688), and pGEX2T-LT- (629–688)] deletion mutants were previously described [35]. pCDNA 3.1-Large T-Ag expression plasmid was described earlier [36].

### Inhibitors and Treatments

Pepstatin A (P 4265), 3-Methyladenine, Rapamycin, and MG-115 were purchased from Sigma-Aldrich. For treatment studies, BSB8 cells were plated in 6-well chamber slides at 80% confluency, transfected with either pCDNA6 Myc-His vector alone or pcDNA6 Myc-His-Bag3-WT expression plasmid. At 24 hours post-transfections, cell culture media were replaced with treatment media containing one of the following reagents: Pepstatin A (100 µM), 3-Methyladenine (5 mM), Rapamycin (50 nM), and MG-115 (30 µM). Cells were treated for 24 hours, and whole cell extracts were prepared, and analyzed by Western blot.

### Reporter Gene Assay

The reporter gene constructs containing the regulatory region of the JCV Mad-1 strain were described previously [37]. Briefly, the Mad-1 (4989 to 480) region was PCR-amplified and inserted into the Bam HI site of the pBLCAT3 vector in early and late orientations. The resulting plasmids were called pBLCAT3-Mad1-Early and pBLCAT3-Mad1-Late. PHFA cells were transfected with these constructs in the presence or absence of expression plasmid encoding Bag3. At 48 h post-transfection, cells were extracted with a series of freeze/thaw cycles, and the CAT activity of the samples was determined as described earlier [37].

### Immunoprecipitation

For the experiments involving the ubiquitination of large T-Ag, BsB8 cells were transfected with expression plasmid encoding Bag3, and whole cell extracts were prepared at 48 h post-transfection. Large T-Ag was immunoprecipitated with a monoclonal antibody (0.4 µg/IP) in immunoprecipitation buffer [25 µl protein A/G beads plus 1%NP-40-TNN buffer (150 mM NaCL, 40 mM tris-HCL pH 7.4, 1 mM DTT, 1 mM EDTA], from either controls or cells overexpressing Bag3. The immunoprecipitation mixture was incubated overnight at 4°C on a rotating shaker. Beads were washed 2 times with 1% NP-40-TNN buffer following 2 more washes with ice-cold PBS. Immunoprecipitated large T-Ag was separated on SDS-PAGE and Western blot performed using anti-ubiquitin antibody. For the experiments involving the in vivo interaction of Bag3 with large T-Ag, large T-Ag and Bag3 was immunoprecipitated with a monoclonal antibody (0.4 µg/IP) and polyclonal antibody (2 µg/IP), respectively, from whole cell extracts prepared from BSB8 cells or U87 MG cells in immunoprecipitation buffer [25 µl protein A/G beads plus 1% NP-40-TNN buffer (150 mM NaCL, 40 mM tris-HCL pH 7.4, 1 mM DTT, 1 mM EDTA]. The immunoprecipitation mixtures were incubated overnight at 4°C on a rotating shaker. Beads were washed 2 times with 1% NP-40-TNN buffer following 2 more washing with cold PBS. Immuncomplexes were separated on SDS-PAGE, and Western blot was performed using either anti-Bag3 or anti-large T-Ag antibodies.

### GST Pull-down Assays

For GST pull-down assays, 2 µg of either GST alone, GST-large T antigen, or its deletion mutants immobilized on Sepharose beads were incubated with 0.5 mg of whole-cell extracts prepared from U 87 MG cells for over-night at 4°C in lysis buffer containing 50 mM Tris-HCl (pH 7.4), 150 mM NaCl, and 0.5% Nonidet P-40. The protein- bead complexes were washed extensively with lysis buffer and resolved by SDS–10% PAGE followed by Western blot analysis using an antibody directed against Bag3 protein.

### siRNAs and Transfections

A specific small interfering RNA (siRNA) (5′-AAGGUUCAGACCAUCUUGGAA-3′) targeting bag3 mRNA and a control, nontargeted (NT) RNA (5′-CAGUCGCGUUUGCGACUGG-3′) were obtained from Dharmacon (Thermo Fisher Scientific, La Fayette, CO). BSB8 cell line was transfected with siRNAs at final concentration of 100 nmol/L using Oligofectamine reagent (Invitrogen). Cells were harvested at 48 h post-transfections.

### JCV Infection

Transfection/infection of cells with the full-length JCV Mad-1 genome was described previously [32]. Briefly, PHFA cells, at a confluence of 1×106 cells per T75-cm tissue culture flask, were co-transfected with JCV-Mad1 genomic DNA in the presence or absence of pCDNA6B plasmid expressing full-length Bag3 using Fugene6 transfection as indicated by the manufacturer (Roche). At 7 days post-infection, cells were trypsinized and harvested for preparation of whole cell protein extract for Western blot analysis.

### Quantitative-PCR (Q-PCR) Analyses of JCV Copy Numbers in Growth Media

Transfection/infection of cells with the full-length JCV-Mad1 genome was performed as described above. The culture medium (containing viral particles) was collected at 7 days post-infection, and after centrifugation at 13,000 rpm for 10 minutes to remove cell debris, supernatants were collected and incubated at 95°C for 10 minutes to inactivate virus. The standard curve was obtained after serial dilution of pJCV, a plasmid containing the whole genome of the JCV Mad-1 strain. All Q-PCR analyses were done by using Lightcycler 480 (Roche). Primers were JCV Q-PCR-forward: 5′-AGTTGATGGGCAGCCTATGTA-3′ and JCV Q-PCR-reverse: 5′- TCATGTCTGGGTCCCCTGGA-3′. The probe for the Q-PCR was 5′−/5HEX/CATGGA TGCTCAAGTAGAGGAGGTTAGAGTTT/3BHQ_1/−3′.
